# Clinical Management of Acromegaly: Therapeutic Frontiers and New Perspectives for Somatostatin Receptor Ligands (SRLs)

**DOI:** 10.3390/medicina58060794

**Published:** 2022-06-13

**Authors:** Alessandro Brunetti, Simone Antonini, Andrea Saladino, Elisabetta Lavezzi, Benedetta Zampetti, Renato Cozzi

**Affiliations:** 1 Endocrinology, Diabetology and Andrology Unit, IRCCS Humanitas Research Hospital, Via Manzoni 56, 20089 Milan, Italy; alessandro.brunetti@humanitas.it (A.B.); simone.antonini@humanitas.it (S.A.); elisabetta.lavezzi@humanitas.it (E.L.); 2 Division of Neurosurgery (NCH 1), Fondazione IRCCS Istituto Neurologico “Carlo Besta”, 20133 Milan, Italy; andrea.saladino@istituto-besta.it; 3 SC Endocrinologia Grande Ospedale Metropolitano Niguarda Milano, Piazza Ospedale Maggiore 3, 20162 Milan, Italy; benedetta.zampetti@ospedaleniguarda.it

**Keywords:** pituitary, acromegaly, therapy, somatostatin, SSA

## Abstract

Somatostatin receptor ligands (SRLs) represent a true milestone in the medical therapy for acromegaly. The first-generation SRLs (FG-SRLs), octreotide and lanreotide, have demonstrated good efficacy in disease control and tumor shrinkage, and are still considered first-line medical therapies. The development of long-acting release (LAR) formulations has certainly improved the therapeutic tolerability of these drugs, although many patients still experience therapy-related burden. As such, new formulations have recently been developed to improve adherence and therapeutic efficacy and more solutions are on the way. In the case of FG-SRL-resistant disease, pasireotide, the only second generation SRL currently available, demonstrated superiority in disease control and tumor shrinkage compared to FG-SRLs. However, its use in clinical practice is still limited due to concern for impairment in glucose homeostasis. In this review, we discuss the news about the present and future role of SRLs in acromegaly, exploring the therapeutical frontiers of this drug class. Moreover, we provide practical guidance on the use of pasireotide, based on the data in the literature and our clinical experience.

## 1. Introduction

Acromegaly is a rare disorder characterized by excessive circulating growth hormone (GH) levels, which in more than 95% of cases is caused by a GH-secreting pituitary adenoma. Due to the wide range of comorbidities associated to GH excess (i.e., hypertension, diabetes type 2, osteoporosis and arthritis, cardiovascular and respiratory disease, increased oncological risk), acromegaly may significantly impair quality of life (QoL) and increase risk of death [1,2].

Universally, the first-line therapy for acromegaly is the surgical removal of the pituitary adenoma via a trans-sphenoidal approach. In specialized centers, surgery results are effective in 80–90% of microadenomas and 50–75% of macroadenomas, with minor efficacy in the case of very large or invasive adenomas [3,4]. When surgery cannot reach resolution or is not feasible, other therapeutic strategies include medical treatment or radiotherapy, although the latter is currently mostly considered a third level therapy to be reserved for selected cases [5].

The goal of medical therapy is to achieve optimal disease control from both a clinical and biochemical perspective, normalizing insulin-like growth factor 1 (IGF-1) levels within an age-specific reference range and limiting the development of disease-related complications. In addition to biochemical control, treatment aims to prevent tumor growth or, ideally, induce tumor shrinkage. Medical therapy includes somatostatin receptor ligands (SRLs), pegvisomant, and dopamine agonists, although the role of the latter is currently considered suitable for patients with minor hormonal hypersecretion.

The release of FG-SRLs, octreotide and lanreotide, dates back to 1980s [6]. Both drugs act as somatostatin analogues with a high affinity for somatostatin-receptor type 2 (SSTR2) and to a lesser extent for SSTR5, inhibiting GH release from pituitary cells and preventing tumor growth. Prospective studies have demonstrated an overall efficacy of 40% in clinical and biochemical disease control, and a significant tumor shrinkage in about 60% of patients, mostly in de novo patients [7,8].

In patients unresponsive to FG-SRLs, the second line of therapy includes pegvisomant (PEGV), a GH receptor antagonist, or pasireotide (PAS), the only second-generation SRLs currently available [5]. PEGV is a genetically engineered GH-receptor antagonist prevalently blocking the GH-induced production of IGF-1 in the liver. In the 2021 update on ACROSTUDY, a 10-year-long global multicenter non-interventional study involving 2221 patients, PEGV showed IGF-1 normalization in 53.7% of the patients after 1 year and in 75.4% after 10 years of treatment. However, due to its mostly peripheral action, PEGV has no impact on pituitary tumor volume, and in ACROSTUDY an increase in tumor size was documented in 7.1% of patients [9].

PAS is a multi-receptor somatostatin ligand approved for acromegaly by both FDA and EMA in 2014, which compared to FG-SRLs expresses a significantly higher affinity for SSTR5 and a lower affinity for SSTR2 [10]. In both phase 3 clinical trials and real-life studies, PAS has proved to be more effective than FG-SRLs in achieving biochemical control and tumor shrinkage [11,12]. It also demonstrated an overall good tolerability profile, with similar side effects to FG-SRL except for an increased risk of developing glucose homeostasis alterations up to overt diabetes. Nevertheless, glucose metabolism alterations occur only in a minority of patients and hyperglycemic events are commonly controlled with standard anti-diabetic therapy [13].

In recent years, more in-depth studies on pharmacokinetic and mechanisms of response to therapy have shed new light on the role of SRLs in the treatment of acromegaly. New formulations have been recently released and highly selective drugs are currently under development. As such, the purpose of this review is to summarize the novelties in the world of SRLs, exploring from new perspectives the present and future role these drugs play in the clinical management of acromegaly.

## 2. Octreotide and Lanreotide: Old Drugs, New Perspectives

The most widely used formulations of FG-SRLs are octreotide in long-acting-release formulation (octreotide LAR), administered via intramuscular injection at a standard dose of 10–30 mg every 4 weeks, and lanreotide Autogel, administered via deep subcutaneous injection at a standard dose of 60–120 mg every 4 weeks.

Despite an overall improved QoL thanks to disease control, patients treated with FG-SRLs still experience reduced QoL because of therapy-related burden [14]. A recent study has shown that around three out of four patients experience gastro-intestinal side effects and/or local site reactions after SRLs injection, impacting on the daily life of more than half of the cases [15]. Therefore, with the purpose of avoiding discomfort of parenteral administration and improving both patients’ QoL and therapeutic adherence, new formulations of FG-SRLs have recently been developed.

An oral formulation of octreotide was approved by Food and Drug Administration (FDA) in June 2020 for acromegalic patients already responsive to injective SRLs. This new formulation was tested in two sponsored randomized controlled trials (CH-ACM-01 and CHIASMA OPTIMAL) including one-hundred-and-fifty-five patients and fifty-six patients, respectively, who were already controlled in FG-SRLs therapy [16,17]. Therapy was administered through oral capsules taken two times a day with water, on an empty stomach, at least 1 h before a meal or at least 2 h after a meal. Both studies demonstrated that about 60% of patients maintained an optimal biochemical response during the follow-up period, which was 52 and 36 weeks, respectively. Recently, a phase 3, randomized, open-label, controlled trial (MPOWERED) [18] has compared responses to oral octreotide and to injective SRLs. The primary outcome was to demonstrate the non-inferiority of the oral formulation compared to the injections (within a margin of difference of 20%). One-hundred-and-forty-six patients initially underwent a 6 month run-in phase using oral octreotide. Among the one-hundred-and-sixteen patients who completed the run-in phase, ninety-two were considered full responders to therapy (IGF-1 < 1.3 × ULN and mean integrated growth hormone < 2.5 ng/mL) and were randomized to either continuing oral octreotide or shifting to injective SRLS. At the end of 9 months of follow up, 91% of patients assigned to oral octreotide maintained a biochemical response, compared with 100% of patients in injective SRLs. As the difference in therapy response did not exceed 20%, non-inferiority of the oral formulation was concluded.

The main limitation of the study was the inclusion in the treatment phase only of patients already responsive to oral octreotide, despite demonstrating the efficacy and safety of the oral formulation. Moreover, despite the presence of substantially overlapping side effects between the two formulations, the satisfaction in patients undergoing oral treatment proved to be significantly superior to injective formulation, as measured with AcroTSQ [19]. Oral octreotide may, therefore, represent an interesting therapeutic strategy in patients with a well-controlled disease under injective FG-SRLS, who have difficulty or are reluctant to parenteral administration.

An octreotide subcutaneous (SC) depot formulation (name CAM2029), is also under development. This formulation uses FluidCrystal technology, allowing both monthly administration and use of thin needles. In a phase 2 trial performed on patients with either acromegaly or functioning neuroendocrine tumors, the SC depot formulation demonstrated to increase octreotide plasma levels more than the IM formulation, with good biochemical control of disease and safety profile [20]. Two phase 3 trials are currently ongoing (ClinicalTrials.gov NCT04076462 and NCT04125836) to assess the long-term (12 month) safety and efficacy of CAM2029. Ideally, the SC depot formulation might be able to achieve therapeutic drug plasma levels avoiding the discomfort of the intramuscular administration of the LAR formulation. Moreover, a phase 1b trial on a 12 week prolonged release formulation (PRF) of octreotide (Debio 4126) has begun, and completion is estimated for the end of 2024 (ClinicalTrials.gov NCT05364944).

In addition, a prolonged release formulation (PRF) of lanreotide is under development, with the purpose of increasing the interval of therapy administration from 4 to 12 weeks. In a phase 2 clinical trial, lanreotide PRF was administered at multiple doses of 180 mg, 270 mg, and 360 mg. The primary endpoint of the study was to find the maximum tolerated dose (MTD), demonstrating good tolerability. Moreover, GH and IGF-1 levels remained substantially stable throughout the study [21]. Therefore, a PRF capable of extending the dose interval appears certainly possible, although further studies are required to better define efficacy and tolerability.

## 3. Pasireotide: Summary of Almost 10 Years of Clinical Experience

### 3.1. Efficacy and Applicability in Clinical Practice

PAS is currently considered in acromegaly therapeutic scenario as a second-line therapy. While for Cushing’s disease the drug is only approved in a short acting formulation, acromegalic patients can benefit from a LAR formulation to be administered at the standard dose of 20–60 mg every 4 weeks. PAS-LAR is suggested for treatment of patients poorly responsive to FG-SRL, especially if there is concern for tumor growth [5]. Indeed, in the phase 3 PAOLA study, PAS-LAR obtained biochemical control of disease in up to 20% of patients previously uncontrolled in FG-SRL therapy, with a further increase to 37% at the end of the extension phase [12,21]. A head-to-head superiority trial comparing PAS-LAR to octreotide LAR on naïve patients with acromegaly confirmed that patients receiving PAS-LAR were 63% more likely to achieve disease control [22]. Regarding tumor shrinkage, clinical trials documented a significant reduction (intended as either >20% or >25%) in up to 80% of treatment naïve patients and about 20% of patients unresponsive to FG-SRLs, with a medium tumor volume reduction of 40% [12]. In real-life settings, patients with uncontrolled disease in FG-SRLs achieved IGF-1 normalization after switching to PAS-LAR in up to 60% of cases, and most of them experienced a significant reduction or even disappearance of headaches [23,24].

In our one-center experience (unpublished data), nineteen acromegalic patients (eight females, 21–69 years old, with macroadenoma, microadenoma, or no evidence of pituitary tumor in 15, 2, 2, respectively) resistant to FG-SRLs at high doses and/or intolerant to pegvisomant were switched to PAS-LAR. Eleven had persistent disease after neurosurgery and two had also undergone radiosurgery (12 and 24 months before starting PAS-LAR). Six complained of acromegalic headache (symptomatic score was 3/3 in 5 and 2/3 in the last). On FG-SRLs, IGF-1 and GH were (mean, range) 193% upper limit normal age-matched range (ULNR) (120–303) and 5.2 ng/mL (0.6–25), respectively. PAS-LAR was injected every 28 days, starting with 40 mg for 3 months, up-titrated to 60 mg if IGF-1 pathologically persisted, or down-titrated to 20 mg if IGF-1 was <50% ULNR. GH and IGF-1 were assessed at 28, 84, and 168 days after starting protocol. Treatment was withdrawn if IGF-1 remained pathologic after 3 months on 60 mg q 28 days. PAS-LAR normalized IGF-1 in 10/19 patients after the first injection and was withdrawn in five unresponsive patients at 6 months. After 12 months, IGF-1 was 74% ULNR (29–133, normal in 9/14) and GH 1.2 ng/mL (0.2–3.9). At the last follow-up (mean 26 months, range 6–60, ongoing dose 20 mg in 3, 40 mg in 7 patients, and 60 mg in 4) IGF-1 was 74% ULNR (22–195, normal in 11/14) and GH 0.7 ng/mL (0.1–2.5). Headache almost disappeared in all patients (in 5/6 after the first injection) and reappeared with pathologic IGF-1 levels after PAS-LAR withdrawal in one irradiated patient. Tumor shrinkage (20–35% of basal volume) was observed in 6/7 evaluated patients without previous irradiation at 6–36 months after the start of PAS-LAR. In two patients, PAS-LAR was withdrawn at 36 and 60 months due to poor compliance in the first, and QTc lengthening in the second, who had started amiodarone treatment.

Recent studies also suggest that the positive effect of PAS in acromegaly may go beyond simple control of disease. A longitudinal retrospective study performed by Chiloiro et al. on patients with resistant acromegaly showed that treatment with PAS reduced the incidence of vertebral fractures (VF), also independently from IGF-1 levels [25]. This finding is even more remarkable considering that other treatments have also confirmed reduction of VF but only in relationship to the control of IGF-1 levels [26]. Authors suggested a possible direct effect of PAS on bone metabolism, although the treatment-specific effects on bone tissues remain unclear and certainly further studies will be needed to confirm and better clarify this association.

PAS may also represent the best therapeutic option for rare types of acromegaly associated with large pituitary tumors, such as X-linked acrogigantism (X-LAG) or aryl hydrocarbon receptor-interacting protein (AIP) mutation positive acromegaly, which are often resistant to FG-SRLs [27]. In a case report of X-LAG acromegaly described by Daly et al., PAS succeeded in inhibiting GH-secretion from tumor culture cells while octreotide did not; unfortunately, a limit of the study was that treatment was only tested in culture cells and not in the clinical setting [28]. In another case series from the same author, two patients with AIP mutation and octreotide-resistant acromegaly achieved optimal control of disease and significant tumor shrinkage when treated with PAS [29].

### 3.2. Treatment-Associated Hyperglycemia: Clinical Management

The use of PAS is still limited in clinical practice by concerns about the development of alterations in glucose metabolism. As such, we considered useful to include in this review a section regarding clinical management of PAS-induced hyperglycemia.

**Figure 1:**
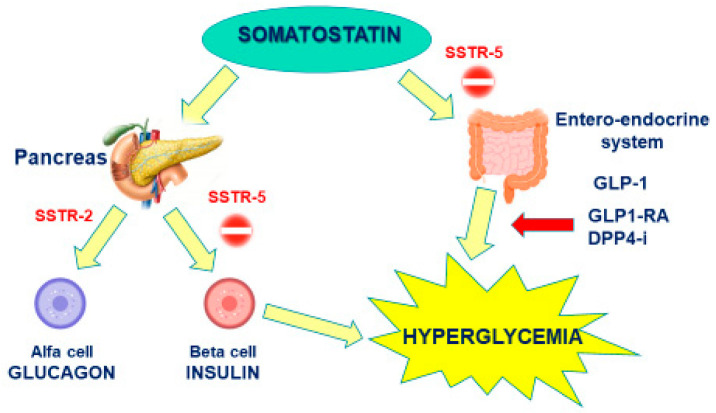
Mechanism of Pasireotide-induced Hyperglycemia.

The pathophysiology underpinning PAS-induced hyperglycemia depends on its higher affinity for SSTR5 than SSTR2. In fact, SSTR5 is highly expressed in pancreatic beta cells, responsible for insulin production, and in entero-endocrine cells is responsible for incretin release (i.e., glucagone-like peptide type 1), while SSTR2 expression prevails in alpha pancreatic cells, mainly responsible for glucagon production. As such, PAS affinity for SSTR5 induces a marked inhibition on both insulin and GLP-1 secretion, with a minor effect on glucagon, whose secretion is instead inhibited by FG-SRLs (Figure 1). A study performed by Henry et al. on healthy volunteers confirmed that a twice-daily subcutaneous administration of 600 or 900 µg of PAS significantly decreased plasma levels of insulin, GLP-1, and glucose-dependent insulinotropic polypeptide, and only slightly affected glucagon secretion [30].

Nevertheless, PAS-induced diabetes mellitus generally involves only a minority of patients. A post hoc analysis performed on the population recruited in the PAOLA study reported that starting an antidiabetic treatment (OAD) was necessary in only 25% of patients, as another 25% was already under diabetic treatment at baseline, and 50% required no specific therapy. Real-life studies show worsening on glucose metabolism or necessity for intensification of OAD in almost 60% of patients, although adding an OAD was required in only one-third of patients and a therapeutically different form metformin was added only in 20% of patients [21]. In our series, HbA1C was 40.6 mmol/mol (29–54) at basal evaluation and no patient was taking OAD. HbA1c was 43.9 mmol/mol (32–66) at 12 months and 43.3 mmol/mol (29–66) at the last follow-up. Glucose metabolism derangement was observed in six patients, with one case of diabetic ketoacidosis (DKA). Metformin was started in four patients and GLP-1 RA in two (in one coupled with insulin).

As such, PAS certainly exerts a worsening effect on glucose metabolism. In few cases, glucose homeostasis derangement can be significant, increasing the risk of DKA and requiring prompt insulin therapy, but in most cases it unlikely progresses to overt diabetes or requires substantial modification of OAD. Several studies have investigated the possible factors contributing to this progression, and the most relevant was the presence of an already altered glucose metabolism at baseline; other possible contributors include increasing age, dyslipidemia and hypertension [31]. Thus, it is essential to assess a complete glucose profile of the patient before starting treatment with PAS and to perform a periodical monitoring of glucose homeostasis.

It has been suggested that in the first three months of treatment, home fasting glucose measurement should be performed once or twice a week in case of normal glucose tolerance at baseline, and daily in case of impaired fasting glucose (IFG) or impaired glucose tolerance (IGT). If no glucose metabolism alteration develops during the first 3 months, home glucose monitoring can be discontinued and only a three month assessment of HbA1C is suggested [13,32]. In patients developing overt diabetes, most common flow-charts suggest metformin as a first-line therapy and, whether abnormal glucose profile persists, a GLP-1 receptor agonist (GLP1-RA) or a dipeptidyl-peptidase-4 inhibitor (DPP4-i).

The choice of these two classes of oral antidiabetic therapy relies on the mechanism of PAS-induced hyperglycemia, as the use of a DPP4-I or a GLP1RA can counterbalance the inhibition on endogenous GLP1 release induced by PAS. The positive effect of incretin drugs on PAS-induced hyperglycemia was confirmed in a recent phase 4 trial, including 249 patients with either acromegaly (190) or Cushing’s disease (59) [33]. Patients started treatment with PAS-LAR 40–60 mg every 28 days (for acromegaly) or PAS 600–900 µg bid (for CD) and underwent an initial 16 week pre-randomization phase of glycemic monitoring. Patients with average fasting glucose ≥126 mg/dL on three consecutive days during the pre-randomization period were randomized 1:1 to incretin-based therapy (sitagliptin (DPP4-I) followed by liraglutide (GLP1-RA) rescue therapy) or insulin for another 16 weeks. Both groups showed a substantially overlap in control of HbA1c and fasting plasmatic glucose (FPG), with even a slight advantage in the incretin group. Unfortunately, patients undergoing DPP4-I or GLP1-RA were not considered separately, and thus is not possible to define the superiority of one treatment over the other; however, GLP1-RA should be theoretically more effective as they replace the inhibited endogenous GLP1, while DPP4-I only extends its half-life [33]. The greater effectiveness of GLP1-RA is suggested also by a study carried out on healthy subjects taking PAS, in which liraglutide proved to be more effective than vildagliptin in improving insulin sensitivity [34].

Noticeably, in most of patients of the study from Sanson et al. [33], no modification of OAD therapy was required throughout the study, and half of patients with acromegaly did not develop significant hyperglycemia at all, confirming that PAS is an overall safe therapy and potential alterations of glucose metabolism should not preclude the use of this drug in real life settings.

### 3.3. Management of Other Adverse Drug Reactions

Aside from alterations in glucose metabolism, PAS has demonstrated both in clinical trials and in real-life settings mostly minor adverse drug reactions (AE). In the PAOLA extension study, cholelithiasis occurred in almost 30% of patients, although not requiring significant intervention except for one case reported of bile duct stone and cholecystitis. In the ACCESS study, evaluating safety of PAS-LAR 40 mg in forty-four acromegalic patients, gastrointestinal symptoms were reported as the most common non-glucose-related AEs: diarrhea in 38.6% of patients, nausea in 27.3%, and abdominal pain in 18.2% [35]. FDA also warned treatment with PAS with possible bradycardia and QT prolongation, recommending caution in subjects with congenital long QT prolongation, uncontrolled or significant cardiac disease, or under treatment with drugs inducing QT prolongation [36]. However, no significant arrhythmias or episodes of severe bradycardia have been reported in larger clinical trials and real-life settings. As such, we do not consider appropriate to perform closer cardiologic monitoring [37].

## 4. SRLs in Combination Therapy with Pegvisomant: Update

Combining FG-SRLs with PEGV is a therapeutic strategy increasingly used in real-world setting. At the moment, expert consensus recommends combination therapy in patients with resistance to monotherapy and concern for tumor growth and impaired glucose metabolism [5].

However, a recent study on French ACROSTUDY population showed that combination therapy was prescribed in almost half of patients treated with PEGV [38]. Interestingly, the study explored the reason underneath the choice of physicians to add directly PEGV to SSA and vice versa, rather than using PEGV monotherapy.

Regarding the addition of PEGV to SRLs, the main reason was the raise of IGF-1 levels in the presence of an aggressive disease or tumor with extrasellar invasion. SRLs and PEGV combination therapy has actually proved to achieve a better biochemical control of disease than SRLs alone [39]. Bonert et al. in their single-center prospective study of fifty-one patients treated with weekly PEGV (40 up to 160 mg/week) plus octreotide LAR or lanreotide Autogel, described 96% of biochemical control rate in patients with both previously well and poorly controlled disease [40]. Moreover, despite that the ACROSTUDY clearly demonstrated that PEGV monotherapy is not significantly associated to tumor growth, the discontinuation of SRLs may reduce control on tumor volume to its pre-treatment size [41].

While adding PEGV to SRLs appears to be a logical therapeutical escalation, it is more difficult to justify the addition of SRLs to PEGV, whose main reasons may be uncontrolled disease in PEGV monotherapy, concerning tumor growth and the reduction of frequency of PEGV injections. In the French ACROSTUDY, authors report that 53.3% of patients with uncontrolled disease achieved IGF-1 normalization after addition of SRLs and that PEGV frequency of administration significantly reduced (16% vs. 44.3% of patients receiving less than seven injections per week) [38]. Interestingly, a medium dose of PEGV never exceeded 20 mg per day in the combination group, suggesting that physicians tend to add SRL therapy when disease is not controlled at the daily PEGV threshold of 20 mg, even though the maximum recommended dose of PEGV is 30 mg daily. Another study interviewing the ACROSTUDY population reports in almost 10% of cases that choice of adding or keeping SRLs together with PEGV therapy was made for control of headaches, for which PEGV monotherapy does not show any benefit [39].

Regarding glucose metabolism, two recent meta-analyses showed an overall neutral effect of combination therapy on fasting glucose and HbA1c with a significant reduction of fasting plasmatic insulin [42,43]. Associating FG-SRLs with PEGV, therefore, might represent a feasible choice in patients with resistant disease and overt diabetes in whom PAS might be not recommended.

In selected cases, a novel therapeutic perspective is represented by the combination therapy of PEGV with PAS-LAR [44]. Potential benefits of this combination may be treatment with PEGV and PAS-LAR may be treatment of resistant acromegaly, a PEGV dose reduction and a reduction of altered glycemic control experienced with PAS monotherapy.

The effect of combination therapy PAS + PEGV was analyzed in the PAPE study, involving sixty acromegalic patients previously controlled with FG-SRLs + medium to high doses of PEGV (medium dose at baseline 134 mg/week). After 12 weeks of follow up, PEGV doses were arbitrarily halved (medium dose 61 mg/week), and patients were either randomized to start PAS-LAR 60 mg together with low dose PEGV if IGF-1 levels had risen above the range after halving PEGV, or to PAS-LAR 60 mg monotherapy if IGF-1 levels were within the reference range. After 24 weeks from baseline, patients in PAS-LAR monotherapy group maintained optimal control of disease in 93% of cases, while in the combination therapy group, PEGV could be further reduced to a medium dose 48 mg/week and even discontinued in 68% of patients. Overall, 12 weeks of PAS-LAR therapy led to a 66% reduction of PEGV dose [45]. In addition, an extension of the PAPE study to 48 weeks showed that more resistant patients reached biochemical control with a longer treatment (67.4% at 24 weeks vs. 71.7% at 48 weeks) but the mean PEGV dose went from 47 to 64 mg/week, although still with a 52% dose reduction from baseline [46]. On the other hand, this study evidenced an increase in the frequency of diabetes from 32.8% at baseline to 68.9% at the end of the study after 24 weeks, underlying that PEGV is not able to avoid a surge in adverse events with PAS-LAR combination therapy. This is probably due to a different mechanism of action, because while PEGV ameliorates insulin sensitivity, PAS worsens glycemic control by inhibiting insulin production in beta-cells and reducing incretin levels, with no direct positive consequences of improved insulin sensitivity [47].

In a 2019 longitudinal study, Chiloiro et al. described the efficacy of PEGV + PAS combination therapy in six patients with active acromegaly despite previous treatment with PAS, PEGV or FG-SRLs + PEGV. The patients of this study had large invasive pituitary adenomas with a mean Ki67 of 3.5% and very high GH levels at baseline. All of them reached biochemical control of disease within the first month of PEGV + PAS combination therapy and one patient experienced a significant reduction in tumor size. Despite the small number of patients and unclear dosage of therapies, this study suggests that PAS + PEGV combination therapy might represent the ultimate pharmacological strategy in patients with very aggressive disease and unresponsive to any other treatment [48].

## 5. Future Perspectives for SRLs

After almost 10 years since the release of the last SRL, research is continuing to explore this drug category and new selective receptor ligands are currently under development.

Paltusotine (former CRN00808) is a highly potent, orally administered, SST2 agonist with a >4000-fold selectivity for SST2 over other somatostatin receptor subtypes. In a phase 1 trial, pharmacokinetic analysis proved an estimated half-life of 22–34 h, supporting a once-daily dose. The drug should be administered in a fasting state as a high-fat, high-calorie meal showed to markedly lower plasma concentrations, although a new spray-dried dispersion (SDD) tablet formulation has proved less sensitivity to food administration than the original capsule formulation [49]. Paltusotine was able to markedly suppress GH-release after GHRH stimulation after one single dose administration and, after a one-week treatment, to reduce IGF-1 levels up to 37%. Treatment-related adverse events were mostly mild and comparable to other SRLs. A particular concern has been raised about sporadic occurrence of non-sustained ventricular tachycardia (NSVT) during the study, although prevalence was similar during both pre-dose and post-dose and kept consistent with the prevalence of NSVT in healthy subjects [49]. Two phase 2 trials have been recently concluded: a single-arm trial evaluating patients with acromegaly and unresponsive to FG-SRLS monotherapy after switching to paltusotine (ACROBAT Edge, ClinicalTrials.gov Identifier: NCT03789656) and a two-arm randomized controlled trial on patients responsive to FG-SRLs (ACROBAT Evolve, ClinicalTrials.gov Identifier: NCT03792555). This latter trial did not reach statistical significance due to the low number of subjects involved (13 patients in total). On the contrary, ACROBAT Edge reached its primary endpoint, that was maintaining IGF-1 stable from baseline to the completion of the 13 week treatment period. Indeed, no significant change in IGF-1 levels was recorded at week 13 compared to baseline and 87% of patients who completed the dosing period achieved an IGF-1 within 1.2 × ULN. No significant adverse events leading to treatment discontinuation were recorded.. A phase 3 study (PATHFINDR1) assessing the efficacy and safety of paltusotine on acromegalic patients previously controlled in FG-SRLs is currently ongoing. Results are expected in 2023 (ClinicalTrials.gov Identifier: NCT04837040).

Somatropim, also known as DG3173 or COR-005, is another SRL showing high affinity to SST2, SST4, and SST5. Somatropin has demonstrated in studies on primary cultures of GH-secreting adenomas to suppress GH release in more tumors than octreotide (10/21 vs. 5/21, respectively) and to reduce GH secretion in 38% (6/16) of tumors that were unresponsive to octreotide [50]. A phase 2 trial on acromegalic patients demonstrated that somatropim administered SC in four ascending doses (100, 300, 900, and 1800 μg) led to a similar reduction in GH levels in comparison to octreotide (300 μg), although complete data have yet to be published (ClinicalTrials.gov Identifier NCT02235987).

In addition, two more selective SST2 agonists are currently in development: ONO-5788 and ONO ST-468. The former demonstrated in studies in vivo on rats to significantly reduce basal and GHRH-stimulated GH [51]; phase 1 trials assessing pharmacokinetics of ONO-5788 on healthy volunteers have ended (ClinicalTrials.gov Identifier: NCT03849872 and NCT03571594), although data are still not available. ONO-ST-468 demonstrated to successfully suppress excessive GH secretion in a GHRH/arginine-induced GH hypersecretion model in the monkey [52], but no studies on humans are registered to date [53].

An overview of emerging treatment strategies for acromegaly is given in Table 1.

## 6. Conclusions

After almost forty years since their release, SRLs still represent a milestone for medical therapy for acromegaly. The development of new formulations has opened up new possibilities, with a view to an increasingly personalized therapy based on the patient’s needs. Moreover, the development of more potent drugs such as PAS has improved the therapeutic outcome and adherence for resistant acromegalic patients, allowing an optimal control in most patients with a single monthly injection. In addition, the possibility of combining these drugs with other therapies makes them particularly suitable for a wide range of applications even in difficult clinical scenarios. In conclusion, the development of SRLs drugs is still progressing, with promising new solutions that may be introduced in future clinical practice.

## Figures and Tables

**Table 1:** Novel SRLs under development.

Drugs	Administration	Dose	Development Stage
New formulations of current drugs			
Mycapssa (Octreotide)	Oral	20–40 mig bid	Approved for clinical use
CAM2029 (Octreotide)	Subcutaneous depot	10–20 mg monthly	Phase 3 (ongoing)
Lanreotide PRF	Subcutaneous	180–360 mg every 12 weeks	Phase 2 (completed)
Debio 4126 (Octreotide)	Intramuscular	Unknown *	Phase 1 (ongoing)
Novel compounds			
Paltusotine	Oral	5–60 mg once daily	Phase 3 (ongoing)
Somatropin	Subcutaneous	Unknown ^§^	Phase 2 (completed)
ONO-5788	Oral	Unknown *	Phase 1 (ongoing)
ONO-ST-468	Oral	Unknown *	Phase 1 (ongoing)

* For these drugs, phase 1 trials are still ongoing so standard therapeutic doses are not available. ^§^ Somatropin has been tested in four doses (100, 300, 900, and 1800 μg) administered one-shot; however, the standard dose and frequency of administration is still unknown.

## Data Availability

The data reported in the study are available from the corresponding author upon reasonable request.
